# Patient-Specific 3D-Printed Low-Cost Models in Medical Education and Clinical Practice

**DOI:** 10.3390/mi14020464

**Published:** 2023-02-16

**Authors:** Zhonghua Sun, Yin How Wong, Chai Hong Yeong

**Affiliations:** 1 Discipline of Medical Radiation Science, Curtin Medical School, Curtin University, Perth 6845, Australia; 2 Curtin Health Innovation Research Institute (CHIRI), Faculty of Health Sciences, Curtin University, Perth 6845, Australia; 3 School of Medicine and Medical Advancement for Better Quality of Life Impact Lab, Taylor’s University, Subang Jaya 47500, Malaysia

**Keywords:** 3D printing, cost, heart, cardiovascular disease, model, medicine, anatomy, pathology

## Abstract

3D printing has been increasingly used for medical applications with studies reporting its value, ranging from medical education to pre-surgical planning and simulation, assisting doctor–patient communication or communication with clinicians, and the development of optimal computed tomography (CT) imaging protocols. This article presents our experience of utilising a 3D-printing facility to print a range of patient-specific low-cost models for medical applications. These models include personalized models in cardiovascular disease (from congenital heart disease to aortic aneurysm, aortic dissection and coronary artery disease) and tumours (lung cancer, pancreatic cancer and biliary disease) based on CT data. Furthermore, we designed and developed novel 3D-printed models, including a 3D-printed breast model for the simulation of breast cancer magnetic resonance imaging (MRI), and calcified coronary plaques for the simulation of extensive calcifications in the coronary arteries. Most of these 3D-printed models were scanned with CT (except for the breast model which was scanned using MRI) for investigation of their educational and clinical value, with promising results achieved. The models were confirmed to be highly accurate in replicating both anatomy and pathology in different body regions with affordable costs. Our experience of producing low-cost and affordable 3D-printed models highlights the feasibility of utilizing 3D-printing technology in medical education and clinical practice.

## 1. Introduction

**Figure 1:**
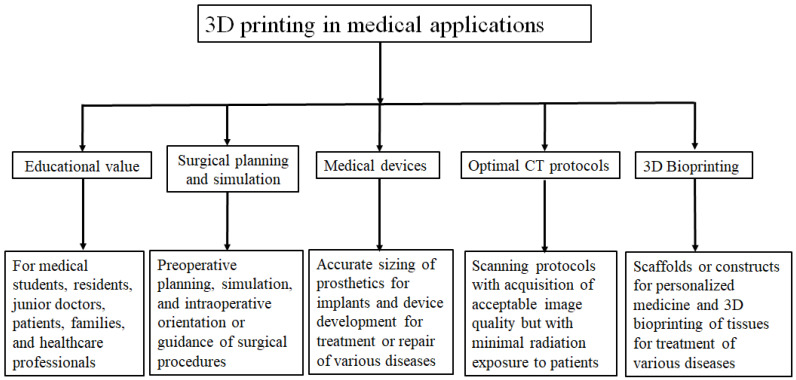
Summary of current medical applications of 3D-printed models. Adapted from Sun et al. [32].

Three-dimensional (3D) printing technology has revolutionized our perception of how advanced technologies contribute to medical education and clinical practice by augmenting the current visualization tools or standard diagnostic or planning approaches used in the different fields of medicine. Patient-specific or personized 3D-printed models derived from medical imaging datasets such as computed tomography (CT), magnetic resonance imaging (MRI) or ultrasound have been increasingly used for medical applications, with research findings proving its value in different aspects [1,2,3,4,5,6,7,8,9,10,11,12,13,14,15,16,17,18,19,20]. Figure 1 summarizes the current medical applications of 3D-printed models. With a generation of high-quality 3D-printed models with a high fidelity of replicating both normal anatomy and pathology, the applications of 3D-printed models have been used in many areas, serving as a valuable additional tool to the current methods [21,22,23,24,25,26,27].

Use of 3D-printed models has been well explored in the maxillofacial and orthopaedics areas and its value in cardiovascular disease and other areas is showing great promise [10,11,12,13,14,15,16,21,22,23,24,25,26,27]. Although promising results are available in the literature, one of the main obstacles to implementing 3D-printing technology on a large scale is due to the relatively high cost and limited access to the 3D-printing facilities [28,29,30]. This includes the software tools used for image processing and segmentation, 3D printers, printing materials, and the process of post 3D printing (such as model cleaning, etc.). The cost per model varies widely ranging from less than USD 100 to more than USD 1000, depending on the purpose of using these models for medical applications (whether they are used for medical education or clinical communication or simulation of surgical procedures or surgical planning) [29,30,31,32]. In this article, we present our experience of producing low-cost and affordable 3D-printed personalized models in medical applications with a focus on cardiovascular disease over the last five years, through collaboration between two international institutions. Our purpose is to share our experience of utilizing a locally available 3D-printing facility at a tertiary institution to print different anatomical models and demonstrate the usefulness of these models in medical education and clinical applications.

## 2. 3D Printing Preparation: Image Post-Processing and Segmentation

It is a standard process to perform image post-processing and the segmentation of CT, MRI and sometimes ultrasound data in a digital imaging and communications in medicine (DICOM) format, using either commercially available software or open source tools to segment the volume data. Mimics (Materialise, Leuven, Belgium), MeVislab (Mevismedical Solutions, Bremen, Germany) and Analyze 12.0/14.0 (AnalyzeDirect, Inc., Lexana, KS, USA) are commonly used commercial software packages for image post-processing and segmentation, while open source tools such as 3D Slicer (Brigham and Women’s Hospital, Boston, MA, USA) and ITK-SNAP (http://www.itksnap.org/pmwiki/pmwiki.php, accessed on 28 January 2023) are also used to create 3D-printed medical models with high accuracy [31]. Of these tools, Mimics is the most commonly used software for 3D printing, in particular in the creation of cardiovascular models, due to its extensive function of segmenting cardiac structures. 3D Slicer, an open source tool, is also commonly used in research publications [31].

**Figure 2:**
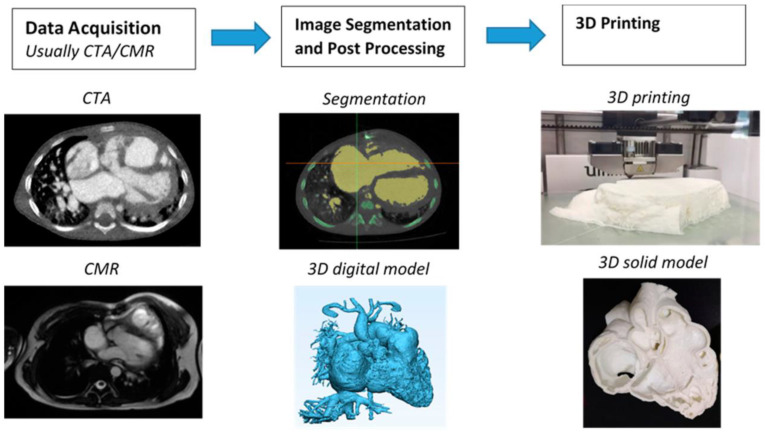
Steps involved in the creation of 3D-printed heart models using Mimics software for image post-processing and segmentation. CTA—computed tomography angiography, CMR—cardiac magnetic resonance, 3D—three-dimensional. Reprinted with permission under the open access from Sun et al. [33].

**Figure 3:**
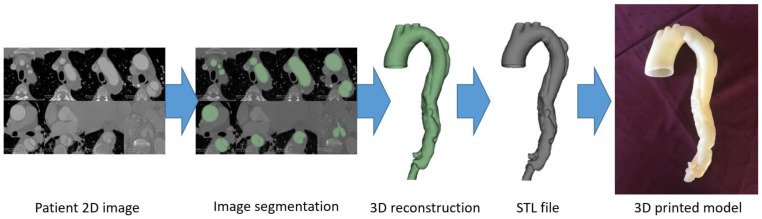
Steps involved in the generation of a 3D-printed aortic dissection model using 3D Slicer. STL—standard tessellation language. Reprinted with permission under the open access from Wu et al. [34].

Figure 2 is a flow chart showing the steps to create a 3D reconstruction model for printing a heart model using Mimics software [33], while Figure 3 is another example showing the steps to create a 3D aortic dissection model using 3D Slicer [34]. There is no standard requirement for choosing the software tools to perform image segmentation, since the final aim is to develop good-quality segmented volume data for 3D-printing purposes. Our experience shows that both commercial and open source tools can achieve the goal of image post-processing and segmentation. 

After image segmentation, the 3D surface model is universally stored in a standard tessellation language (STL) format and sent to a 3D printer to print a physical model. Although an STL file can be printed at this stage, another step always involves using computer-aided design (CAD) software to post-process or refine the segmented surface model before proceeding to the final stage of 3D printing. The segmented geometry model usually has a rough surface which is commonly seen in cardiovascular models due to the complex anatomical structures; thus, the use of CAD manipulations is necessary to optimize the 3D surface model, such as wrapping or smoothing the surface of the 3D object by removing any artefacts or unwanted structures from the source data, and enhance the 3D model to match the anatomy and pathology as shown in the original source data [1]. Commonly used CAD software tools for medical modelling include Meshlab (Italian National Research Council, Pisa, Italy), Meshmixer (Autodesk Inc., San Rafael, CA, USA) and Blender (Blender Foundation, Amsterdam, The Netherlands).

The final step is the 3D printing of the physical models. Fused deposition modelling (FDM), stereolithography (SLA), selective laser sintering (SLS) and polyjet printers are commonly used in printing models for medical applications [1,32,35]. The following section provides details of the available 3D printers and printing materials that were used to print our personalized models.

## 3. 3D Printing Facility: 3D Printers and Printing Materials

The 3D-printing laboratory was established in Taylor’s University in 2018 and it was primarily used for teaching purposes. The laboratory was equipped with multiple fused deposition 3D printers and digital light processing 3D printers. These printers are capable of printing 3D models with numerous materials such as polylactic acid (PLA), acrylonitrile butadiene styrene (ABS), polyethylene terephthalate glycol (PETG), high impact polystyrene (HIPS), polyvinyl alcohol (PVA), nylon, thermoplastic polyurethane (TPU), polyurethane (PU) and polymethacyrlate (PMMA).

Table 1 is a summary of the models that were created over the last five years for different medical applications. A total of 50 models were generated with the use of different types of printers and printing materials to suit medical applications. These models were printed at a 1:1 life size ratio, thus replicating the true anatomical structures including normal anatomy and pathology when compared to the original source imaging data. In the following sections, we share our experiences of using these models for a range of applications.

## 4. Usefulness of 3D-Printed Models in Cardiovascular Disease

More than 70% these models were 3D-printed heart and vascular models with investigations focusing on congenital heart disease (CHD) and coronary artery and aortic aneurysm and dissection studies. Applications of these models ranged from medical education to pre-surgical planning and the simulation of cardiac procedures, as well as the development of optimal cardiovascular CT scanning protocols [31,32,33,34].

### 4.1. 3D-Printed CHD Model Accuracy

**Figure 4:**
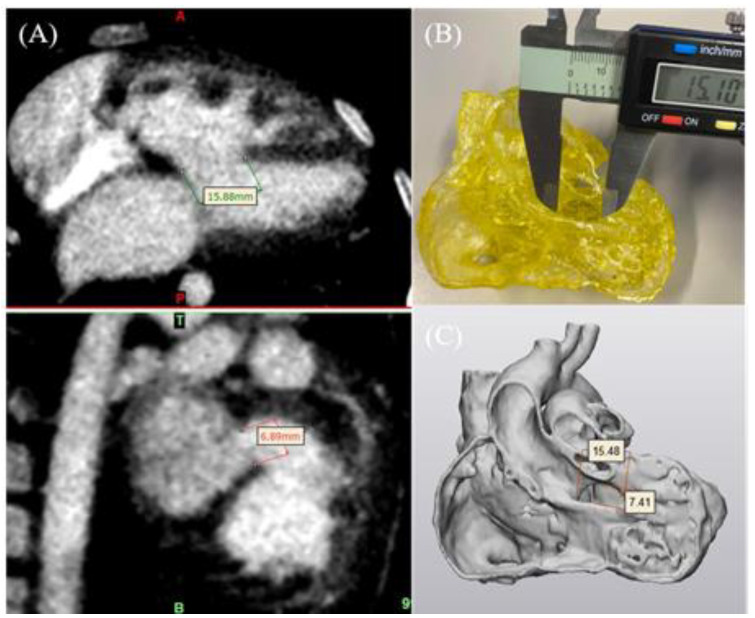
3D-printed model accuracy. (**A**) CT imaging data in coronal and sagittal views (left top and bottom images) to measure the VSD on source imaging data. (**B**) Measurement of the VSD in the 3D-printed model using a digital calliper. (**C**) STL file measurement of the VSD in 3-Matic. VSD—ventricular septal defect. Reprinted with permission under the open access from Lee et al. [22].

Model accuracy comprises an essential component in 3D printing as the physical models must accurately replicate normal anatomy and pathology when compared to the original source images so that they can be reliably used for different applications. Our studies and others have confirmed that these models are highly accurate with differences of less than 0.5 mm between the 3D-printed models and original source images (Figure 4) (Table 2) [22,23,24,25,26,27,36]. These study results showed that the 3D-printed models are highly accurate with less than 0.5% deviation in diameter measurements between the 3D-printed models and original source images; hence, the difference is considered negligible [37,38].

### 4.2. 3D-Printed CHD Models in Medical Education

**Figure 5:**
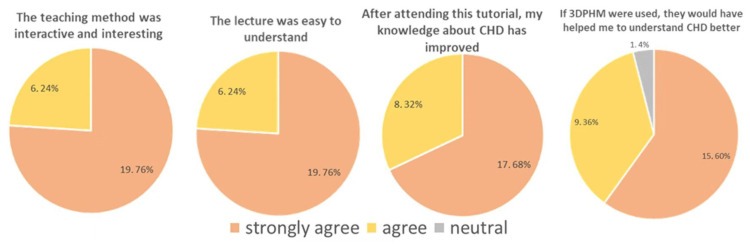
Survey response (number of students, percentage) of the control group with regard to the education session. 3DPHM—3D-printed heart model. Reprinted with permission under the open access from Lau and Sun [49].

**Figure 6:**
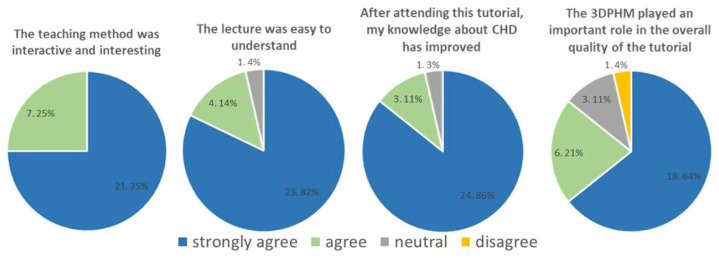
Survey response (number of students, percentage) of the 3D-printing group with regard to the education session. 3DPHM—3D-printed heart model. Reprinted with permission under the open access from Lau and Sun [49].

**Figure 7:**
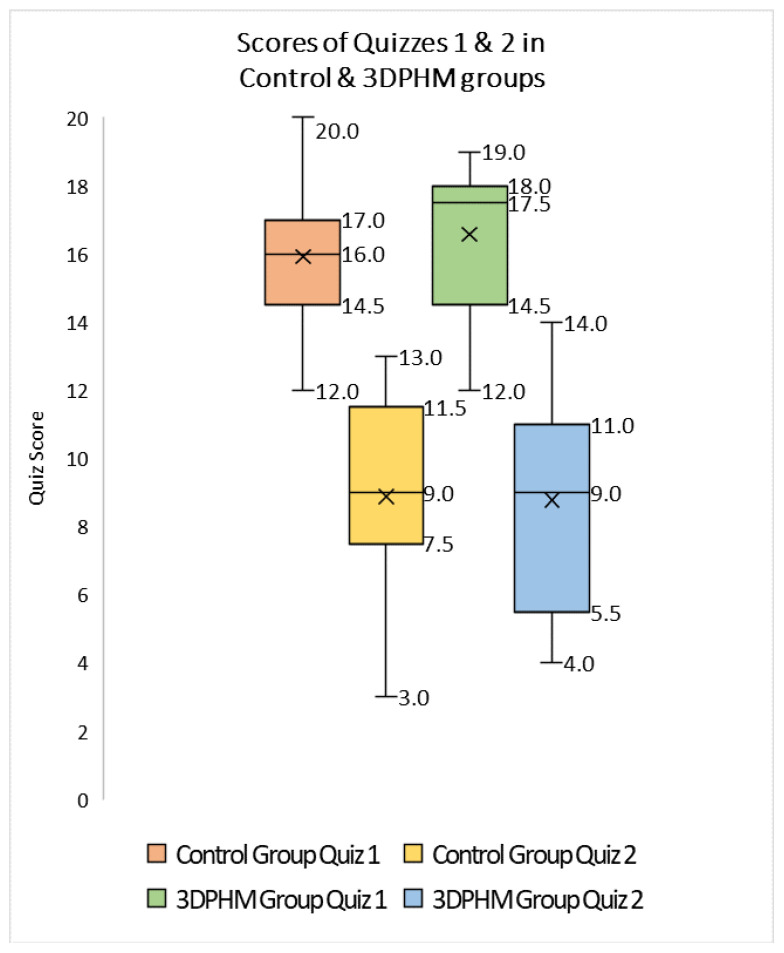
Boxplot of the scores (out of 20) achieved by 3D printing and control groups in Quiz 1 and Quiz 2. 3DPHM—3D-printed heart model. Reprinted with permission under open access from Lau and Sun [49].

3D-printed CHD models represent the most common application of cardiovascular disease according to several randomized controlled trials and cross-sectional studies [22,39,40,41,42,43,44]. This is most likely due to the difficulty in fully comprehending the cardiac anatomy and congenital anomalies associated with CHD conditions on traditional 2D or 3D visualizations. 3D-printed CHD models enhanced medical students’ knowledge of CHD compared to the current teaching methods using diagrams or cadavers or standard image visualizations [39,40,41,42,43,44,45,46,47,48]. We created four CHD models and recently reported our experience of exploring the educational value of 3D-printed CHD models in second and third year medical students (*n* = 53) with regard to their understanding and learning of CHD [49]. Twenty-five students were provided with 2D cardiac CT images and 3D digital models, while 3D-printed models were offered to 28 students in the 3D-printing group as an additional component. Four types of CHD were presented to these medical students who completed an online quiz at the end of the session and another online quiz 6 weeks later, with the aim of determining the value of 3D-printed CHD models in immediate and long-term knowledge retention. The results showed that more students in the 3D-printing group strongly agreed that 3D-printed models improved their understanding and knowledge about CHD when compared to the current methods, although this did not reach statistical significance (*p* = 0.16–0.99) (Figure 5 and Figure 6). There were no significant improvements in both immediate knowledge and long-term knowledge retention with the use of 3D-printed CHD models, despite slightly better scores obtained in the 3D-printing group than in the control group (Figure 7) [49].

**Figure 8:**
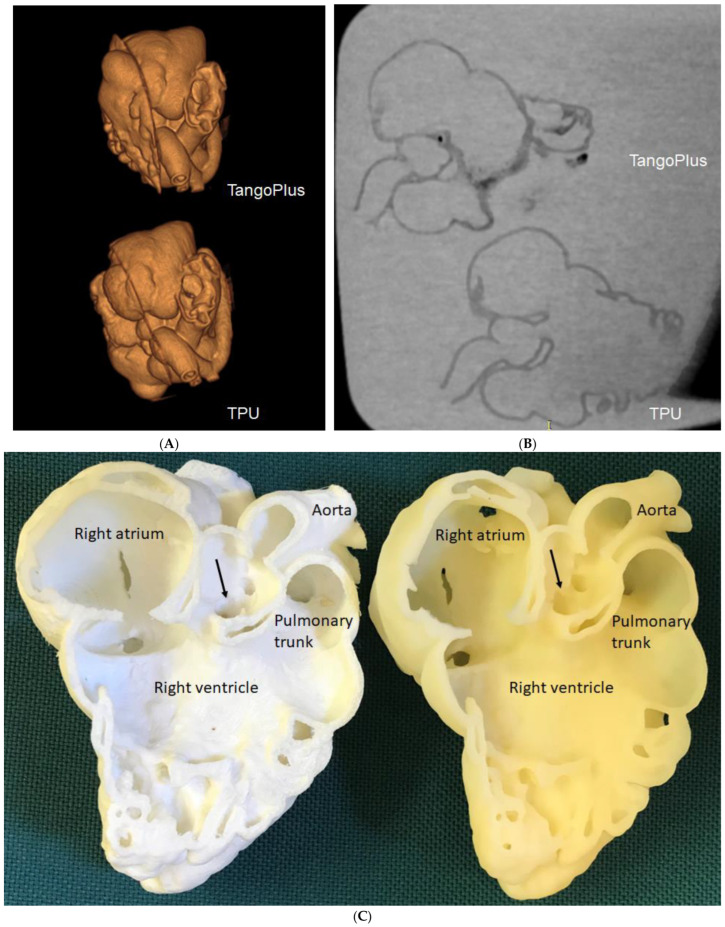
3D-printed congenital heart disease models with use of different materials for comparison of model accuracy. (**A**): 3D CT volume rendering of the 3D-printed models showing similar anatomical details. (**B**): 2D axial CT views of the 3D-printed models. (**C**): Inside view of cardiac chambers and aortic structures on both models. The white model was printed with TPU, while the yellow model was printed with TangoPlus. Arrows refer to subaortic ventricular septal defect. Reprinted with permission under the open access from Sun [51].

One of our studies showed that a low-cost CHD model printed with a relatively cheap material produced a similar clinical value as the high-cost model [26,50,51]. Figure 8 shows a 3D-printed CHD (double outlet right ventricle) model using low-cost thermoplastic polyurethane (TPU, USD 25) and relatively high-cost TangoPlus (USD 200) materials. CT scans of the models showed excellent correlation between the two models in terms of dimensional measurements in different anatomical locations. Both models achieved the same scores ranked by clinicians from the aspects of clinical value in medical education and preoperative planning [50]. Our heart models printed with the TPU materials showed similar values and applications to other studies [40,43].

### 4.3. 3D-Printed CHD Models in Preoperative planning

Another focus of our 3D-printed CHD models was their use in the assessment of pre-surgical planning and the simulation of complex cardiac procedures when compared to standard image visualization and virtual reality (VR). We used the same four 3D-printed CHD models as developed in our previous study [49] and compared their clinical value with VR in both medical education and the preoperative planning of CHD among 29 participants with different medical backgrounds (cardiologists, radiologists, sonographers and radiographers) [52]. Both 3D-printed CHD models and VR were scored useful in displaying CHD anatomy and pathology, although 3D-printed models were found better; while VR was ranked more useful for medical education (for medical students and junior physicians) about CHD and preoperative planning, with no significant differences reached between these modalities in the assessment areas. Twenty-two (76%) participants indicated the usefulness of VR and 3D-printed CHD models to increase a surgeon’s confidence in CHD surgeries, while 72% of participants indicated VR and 3D-printed CHD models offered additional value compared to standard medical imaging visualizations. A subgroup analysis of the participant’s responses between physicians/doctors and technicians/non-doctors did not show significant differences in the clinical value between VR and 3D-printed CHD models (Table 3) [52].

### 4.4. 3D-Printed Coronary Artery Models

**Figure 9:**
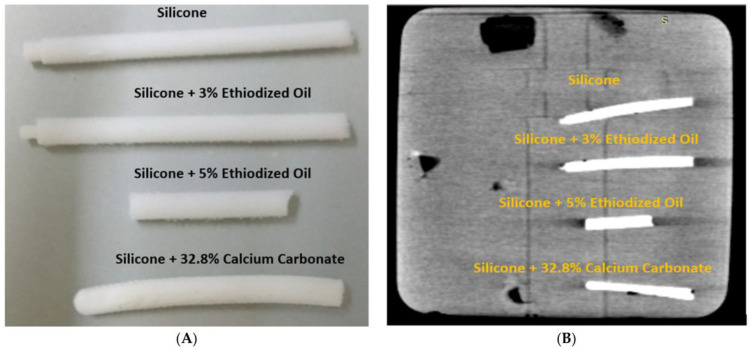
Creation of simulated calcified plaques. (**A**): Different material compositions to simulate calcification in the 3D-printed mould. (**B**): CT image of these materials with measured CT attenuation being 450 HU, 600 HU, 900 HU and 800 HU, corresponding to silicone, silicone + 3% ethiodized oil, silicone + 5% ethiodoized oil and silicone + 32.8% calcium carbonate, respectively. The combination of silicone + 32.8% calcium carbonate was selected to produce 800 HU attenuation, representing highly calcified plaques. Reprinted with permission under the open access from Sun et al. [53].

**Figure 10:**
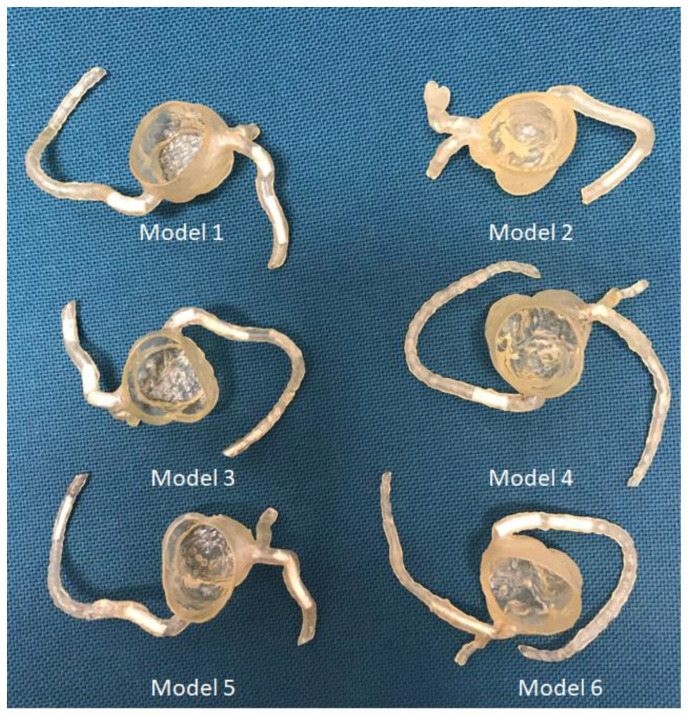
3D-printed coronary artery models with designed 3D-printed calcified plaques inserted into the coronary artery branches to simulate high calcification. Reprinted with permission under the open access from Sun et al. [53].

**Figure 11:**
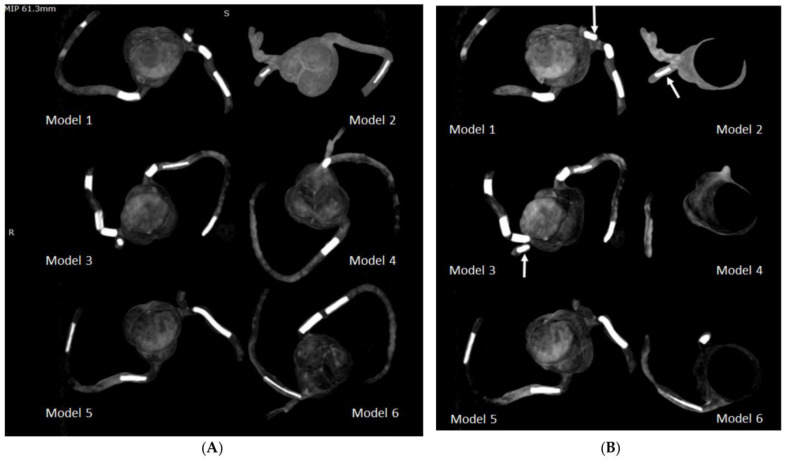
Maximum-intensity projection (MIP) images showing the calcified plaques in six 3D-printed coronary models. (**A**): Coronal MIP view showing these calcified plaques. (**B**): Oblique MIP view showing the plaques more clearly in the left circumflex coronary artery (arrows) in Model 1 (plaque 3), Model 2 (plaque 6) and Model 3 (plaque 11). Reprinted with permission under the open access from Sun et al. [53].

We created six coronary artery models replicating normal coronary artery branches and coronary stenosis. In addition, we designed calcified plaques to simulate high calcification in the coronary arteries for the investigation of optimal CT protocols with a minimization of the blooming artifacts associated with extensive calcification. Figure 9 shows the approach to develop 3D-printed calcified plaques after testing four compositions of different materials, while Figure 10 shows the insertion of these simulated calcified plaques in these 3D-printed coronary artery branches. A coronary CT scan was performed on the 3D-printed coronary artery models with a clear demonstration of the calcified plaques that were placed in the coronary arteries (Figure 11). Our developed 3D-printed models with a simulation of high calcification allows for the investigation of optimal coronary CT protocols to improve the visualization of coronary lumen in the presence of high calcification or coronary stenting [53,54,55]. There are a few studies reporting the development of 3D-printed coronary models and their main applications focus on the treatment of complex coronary anomalies with the aid of 3D-printed models [54,56,57]. Our study further advanced the application of 3D-printing technology to optimize coronary CT protocols.

### 4.5. 3D-Printed Aorta Models

**Figure 12:**
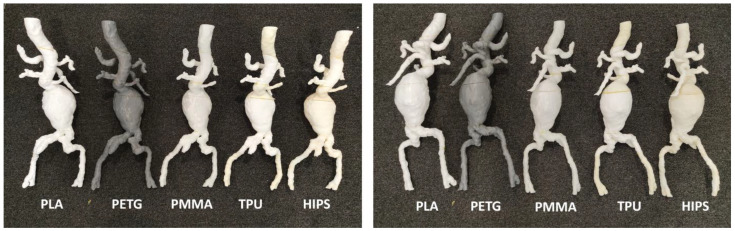
3D-printed abdominal aortic aneurysm models with use of different materials (left image: posterior view, right image: anterior view). These models were used as a pilot test to identify the appropriate material to develop vascular models for the simulation of EVAR procedures. EVAR—endovascular aneurysm repair. HIPS—high impact polystyrene, PLA—polylactic acid, PETG—polyethylene terephthalate glycol, PMMA—polymethacrylate, TPU—thermoplastic polyurethane.

**Figure 13:**
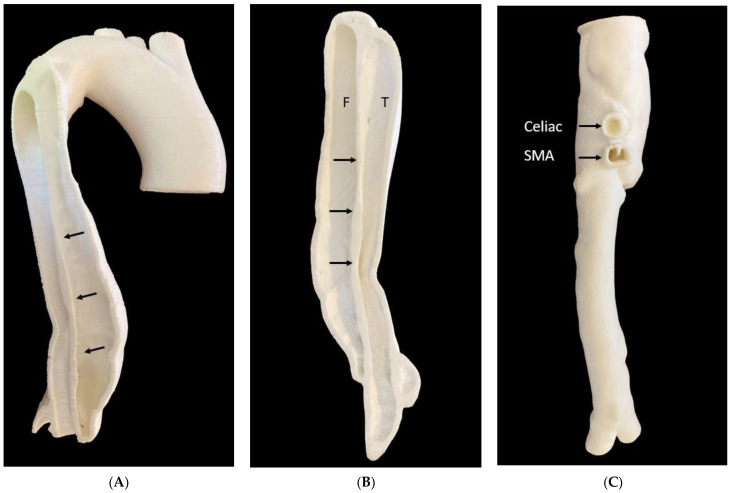
3D-printed type B aortic dissection model with use of thermoplastic polyurethane material. (**A**): Internal view of the main part of the 3D model with aortic lumen separated into true and false lumen by an intimal flap (arrows). (**B**): The other piece of the 3D model with a demonstration of true and false lumens. Arrows refer to the intimal flap. (**C**): Lower part of the 3D model with abdominal aorta and main branches (celiac and SMA) and common iliac arteries. F—false lumen, T—true lumen, SMA—superior mesenteric artery.

**Figure 14:**
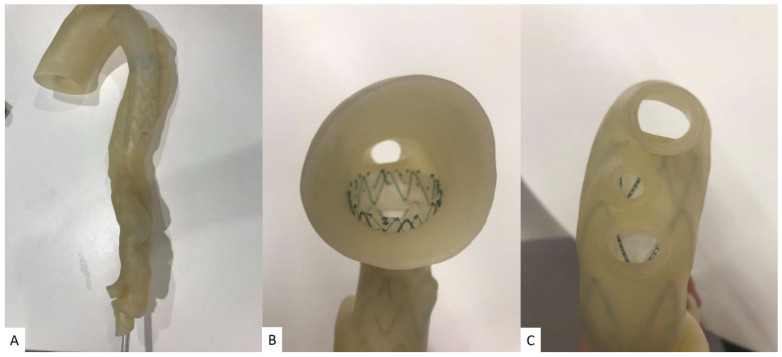
Stent graft deployed in 3D-printed model. The aortic model was printed with soft and elastic Visijet CE-NT A30 (USD 600) with properties similar to normal aorta. (**A**): Deployed stent graft visible through model wall. (**B**): Axial view from proximal aortic arch. (**C**): Caudal view down aortic arch vessels. Reprinted with permission under the open access from Wu et al. [34].

We printed six aorta models based on CT angiographic images to simulate the endovascular aortic aneurysm repair (EVAR) of abdominal aortic aneurysm (AAA) or type B aortic dissection [58,59,60,61]. EVAR is a less invasive procedure commonly used for the treatment of AAA and aortic dissection with lower risks of complications or mortality than open surgery [62,63]. The 3D-printed aorta models (five AAA models) served as a useful tool not only for the simulation and planning of EVAR in complex aneurysm cases, but also for the development of optimal CT scanning protocols, since routine CT angiography follow-up is commonly performed in patients following EVAR treatment [32,64]. Figure 12 shows 3D-printed AAA models with the use of different materials with the aim of simulating EVAR procedures. Figure 13 is an example of a 3D-printed model with the use of low-cost materials showing type B aortic dissection, while Figure 14 is another example of a 3D-printed aorta model using the same dataset but printed with high-cost materials for the simulation of EVAR procedures and investigation of optimal CT protocols [34]. Our purpose of testing different printing materials for the simulation of EVAR procedures is similar to what Torres and colleagues did, but their study participants were vascular residents [58], while our participants will be interventional radiologists or residents. Furthermore, their 3D-printed aneurysm models cost from EUR 120 to 475 per model, which is more expensive than ours.

## 5. 3D-Printed Tumour Models

Personalized 3D-printed models are shown to play an important role in enhancing a viewer’s understanding of the complex anatomy and spatial relationship between tumours and surrounding anatomical structures, with studies reporting its clinical value in surgical training and planning, and the operative simulation of various tumours [6,7,8,9,19,20,37,65]. We have printed several models of different types of tumours from CT and MRI datasets, with the aim of exploring the usefulness of 3D-printed tumour models in preoperative planning when compared to the current approaches based on image visualizations.

### 5.1. 3D-Printed Breast Cancer Model

**Figure 15:**
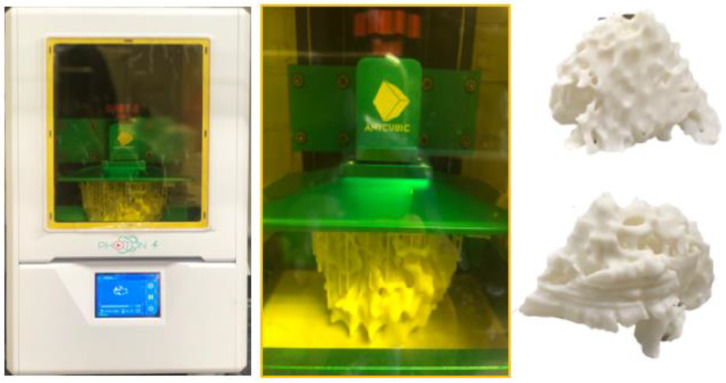
Fabrication of the hollow fibroglandular models using the Anycubic Photon S high-resolution 3D digital light processing printer. The thickness of the wall is 2.0 mm. Reprinted with permission from Sindi et al. [66].

**Figure 16:**
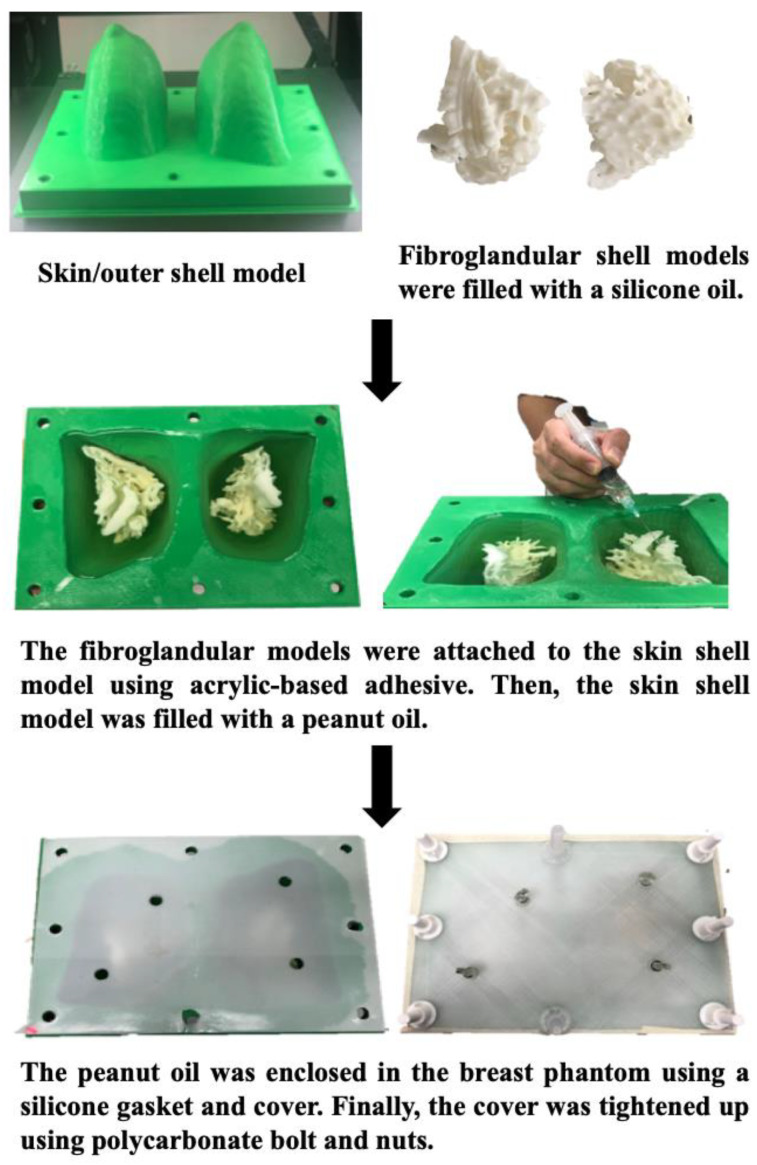
Flow chart showing 3D construction of the breast phantom. 3D printing was used to create the hollow shells for skin and fibroglandular regions. Fibroglandular and adipose tissues were simulated using silicone and peanut oils, respectively. Reprinted with permission from Sindi et al. [66].

**Figure 17:**
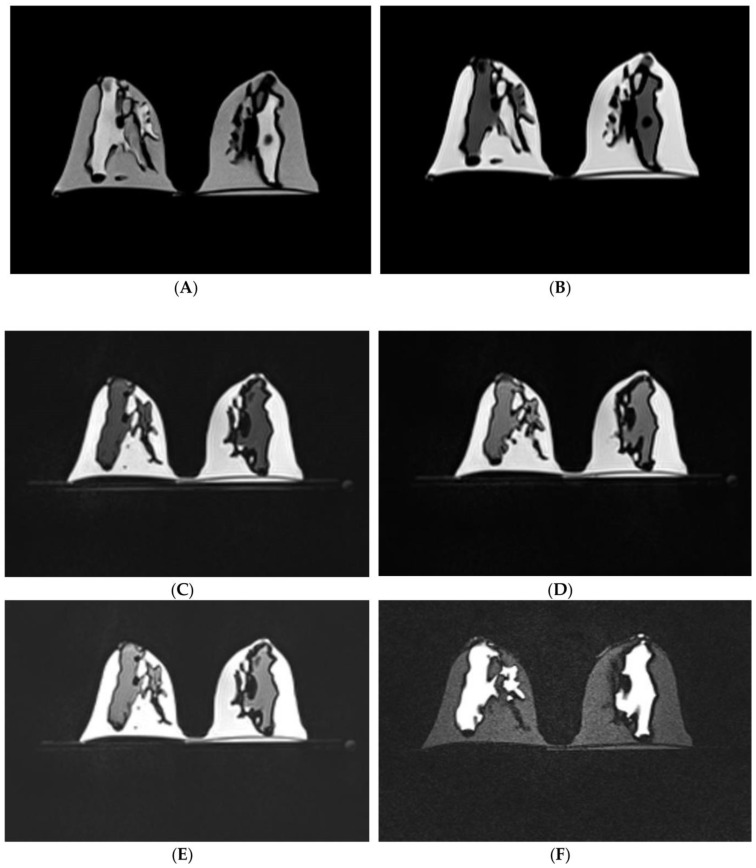
MR images of 3D-printed breast model with use of six different scanning sequences. (**A**) Non-fat-suppressed TSE (T2WI); (**B**) non-fat-suppressed TSE (T1WI); (**C**) non-fat-suppressed TSE SPACE (T1WI); (**D**) fat-suppressed TSE SPACE (T1WI); (**E**) fat-suppressed TSE SPACE SPAIR (T1WI); (**F**) fat-suppressed IR/PEF-TIRM (T2WI). T1WI—T1 weighted imaging, T2WI—T2 weighted imaging, TSE—turbo (fast) spin echo, SPACE—sampling perfection with application optimized contrast using different flip angle evolution, SPAIR—spectral attenuation inversion recovery, IR—inversion recovery, PFP—partial Fourier phase, TIRM—turbo inversion recovery magnitude. Reprinted with permission under the open access from Sindi et al. [67].

We developed a patient-specific 3D-printed breast model from a normal breast MRI scan and identified suitable materials simulating MR imaging features of adipose and fibroglandular tissues [66,67]. First, we used 3D-printing technology to create the hollow skin and fibroglandular region shells using tissue-mimicking materials. Then, we tested five materials (agarose gel, silicone rubber with/without fish oil, silicone oil, and peanut oil) and measured their T1 relaxation times on a 3T MRI scanner. The results showed that silicone oil’s T1 relaxation time was similar to that of fibroglandular tissue, while peanut oil’s T1 relaxation time was similar to that of adipose tissue. Hence, silicone oil and peanut oil were injected into the 3D-printed fibroglandular model and skill shell model, respectively (Figure 15 and Figure 16). Furthermore, we scanned the 3D-printed model with six different MR sequences including fat- and non-fat suppressed sequences to perform quantitative measurements of breast volume, fibroglandular tissue volume and the percentage of breast density between these two different scanning sequence groups [66]. Quantitative measurements of breast fibroglandular tissue volume and the percentage of breast density on fat-suppressed sequences were significantly higher than those measured on non-fat suppressed sequences (*p* < 0.05), although there was no significant difference in breast volume measurement (*p* = 0.529) [67]. Figure 17 shows MR images of the 3D-printed breast model with six different imaging sequences.

There are only a few studies available in the literature regarding the development of 3D-printed breast models for use in the medical imaging area [68,69,70], while research on the assessment of breast density with the use of a realistic 3D-printed model is lacking. Our 3D-printed breast model can be used to identify optimal breast MR scanning parameters for the quantitative analysis of breast density.

### 5.2. 3D-Printed Lung Cancer Model

**Figure 18:**
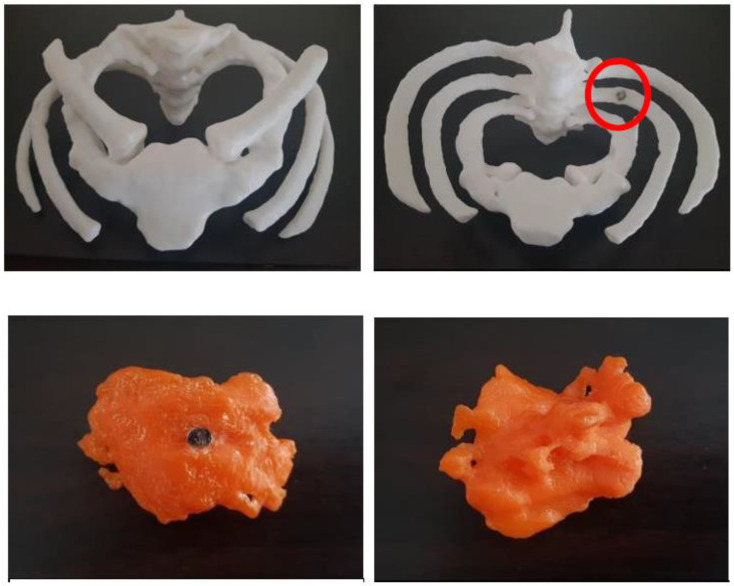

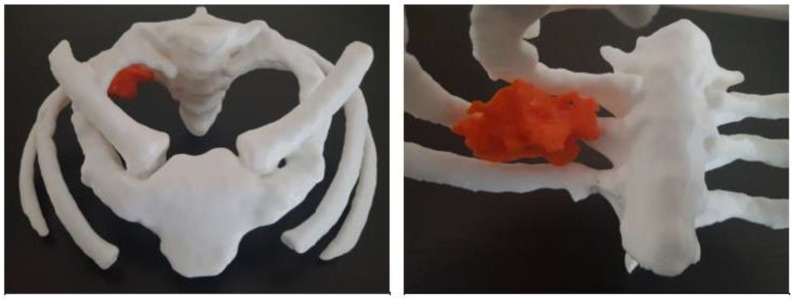
3D-printed Pancoast lung cancer model and bones. (**Top**) row: frontal and bottom views with magnet (circled). The model was printed with polylactic acid. (**Middle**) row images: (**Top**) view of 3D-printed tumour with magnet and bottom view. (**Bottom**) row images: 3D-printed model with tumour and bony structure added together (frontal and superior views of the exact tumour location in the right lung apex). Reprinted with permission from Yek et al. [72].

We printed a lung cancer model based on CT images of a patient diagnosed with a Pancoast tumour, which is located in the lung apex. Surgical resection of Pancoast tumours could be very challenging because of their invasion into surrounding structures such as ribs, vertebrae, blood vessels and muscles [71]. We reviewed two cases of Pancoast tumours and chose an operable case with bones and the tumour printed using different materials (Figure 18). The models were presented to two cardiothoracic surgeons with more than 10 years of experience for the evaluation of the usefulness of 3D-printed models as a preoperative tool. Participants agreed that the 3D-printed model offered a better representation of the exact tumour location relative to bones when compared to standard CT images. The model was considered to have potential value in assisting operation and facilitate communication between team members. It was also found to be extremely useful in medical education [72]. Studies reported the clinical value of using 3D-printed models in improving surgical safety and patient’s understanding of surgical resection of lung cancer [73,74], but at the cost of USD 1000 printing per model [73].

### 5.3. 3D-Printed Renal Cell Carcinoma Model

**Figure 19:**
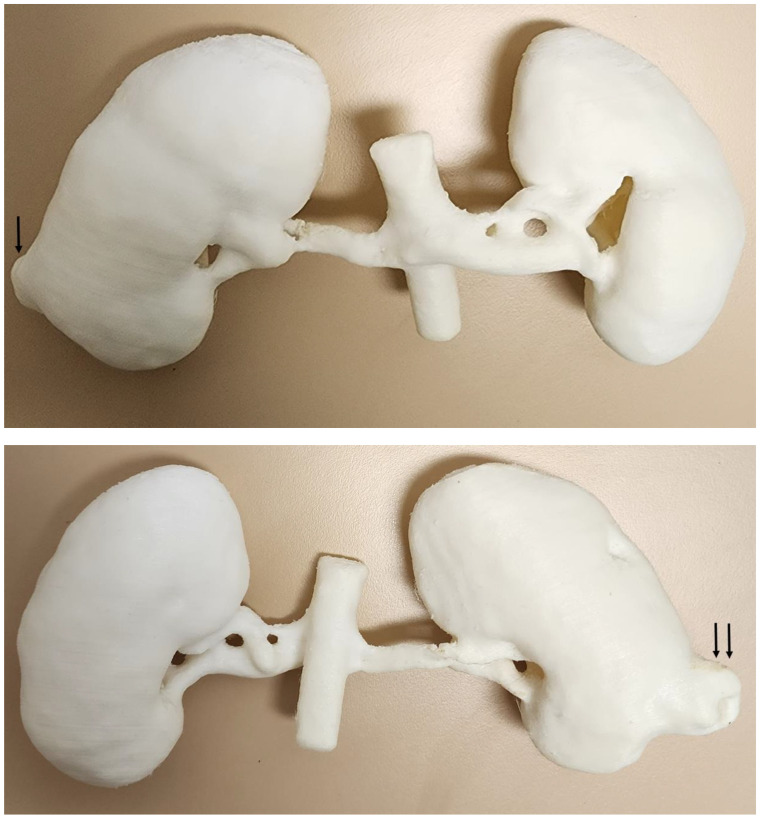
3D-printed kidney model with a tumour at the lower pole of right kidney (arrows). The model was printed with thermoplastic polyurethane material. (**Top**) image: frontal view of the 3D-printed model does not show the tumour (arrow) clearly due to its location on the posterior aspect of right kidney. (**Bottom**) image: posterior view of the 3D-printed model shows the tumour (arrows) clearly.

3D-printing technology is increasingly used in printing kidney models for renal disease with research findings showing its clinical value in the preoperative planning and simulation of renal disease, education of junior surgeons, enhancement of operative skills for senior surgeons, as well as the facilitation of interdisciplinary communication and decision making in terms of the management of patients with renal cell carcinoma (RCC) [6,7,75,76,77]. We chose a case with low-grade renal cell carcinoma on the inferior pole of right kidney and printed the model with TPU (Figure 19). Measurements of dimensional accuracy at different anatomical locations did not show significant differences between the 3D-printed model, original CT images and STL file. The 3D-printed model was presented to five urologists with 5–20 years of experience in the surgical treatment of RCC. All participants agreed that the 3D-printed model could facilitate preoperative planning, and believed that it could reduce intra-operative complications. They also agreed that a 3D-printed model could be used for the training of inexperienced surgeons and for patient education and patient–clinician communication [78]. Our developed low-cost model is suitable for medical education and patient communication, while for clinical applications such as the pre-surgical planning of RCC resection, 3D models printed with multi-colour materials are preferable, as shown by a recent systematic review [79], despite the relatively high cost (between USD 400 and 1000).

### 5.4. 3D-Printed Pancreatic Cancer Model

**Figure 20:**
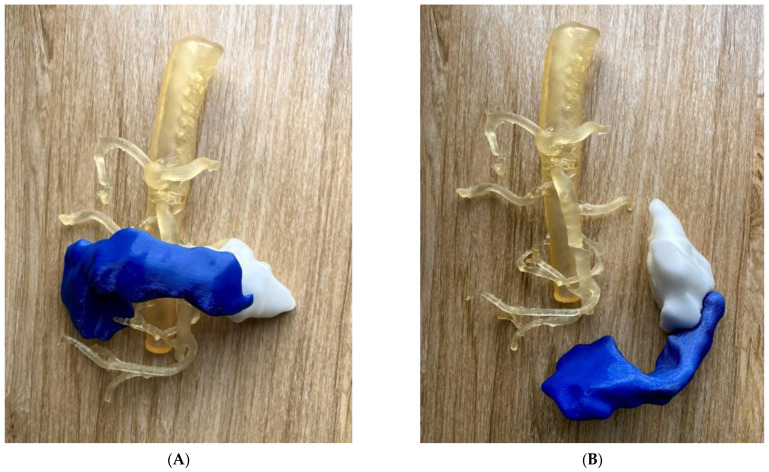

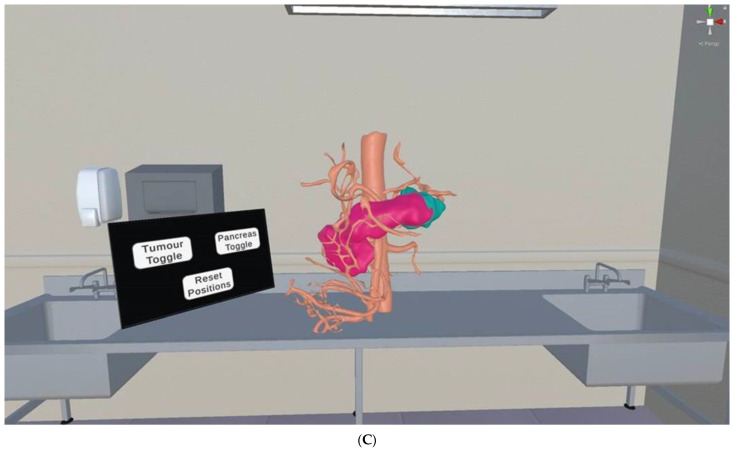
Clinical value of 3D-printed pancreas model in comparison with virtual reality (VR). (**A**): 3D-printed pancreatic model and abdominal aorta and arterial branches were placed together. (**B**): 3D-printed pancreatic model and abdominal aorta model separately. (**C**): VR screenshot of 3D view of pancreatic tumour (green) and pancreatic parenchyma (pink). The blue colour refers to a normal pancreatic parenchyma, and the white colour indicates the pancreatic tumour in images (**A**,**B**).

The use of 3D printing in pancreaticobiliary disease is only reported in a few case studies with results showing that 3D-printed models improve the outcome of pancreaticobiliary surgeries by enhancing the understanding of the operation process and serving as a training tool [5,20,80,81]. We printed a pancreatic cancer model along with abdominal aorta and main arterial branches and also created VR views for comparison with 3D-printed models with regard to their value in the preoperative planning of pancreatic tumours (Figure 20). We invited six participants (four pancreatic surgeons, one surgical resident and one gastroenterologist) to provide their opinions on the clinical value of both 3D-printed models and VR in the preoperative planning of pancreatic tumour resection. All participants agreed that both the 3D-printed model and VR offered better spatial awareness between the pancreas and surrounding vessels, and helped the planning of complex surgery when compared to the original CT images. Five out of six participants considered that VR was more useful than the 3D-printed model in the preoperative planning of pancreatic tumour resection. Further studies with the inclusion of more participants, especially novice surgeons, are needed to validate the clinical value of 3D-printed pancreatic models in preoperative planning or skill improvement. Our preliminary findings are consistent with others [80,81], although future studies should include more cases and participants to allow robust conclusions to be drawn.

### 5.5. 3D-Printed Biliary Cyst Model

**Figure 21:**
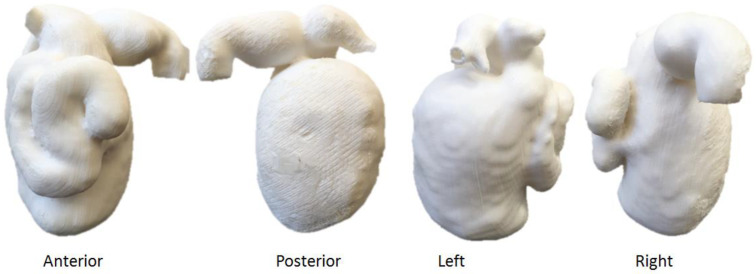
3D-printed biliary cyst and bile ducts from different viewing angles.

Application of 3D-printing technology in biliary disease is limited as most of the current reports are focused on hepatic disease such as hepatocellular carcinoma or liver transplant [2,8,9]. We generated a 3D-printed model from a case with a rare and huge biliary cyst in the common bile duct (Figure 21) [82]. Right and left hepatic ducts, and common bile duct including the cyst, were printed with 3D-printed model scanned on a 64-slice CT scanner. CT images of the 3D-printed model were used to measure dimensions of these biliary trees for comparison with an STL file and 3D-printed model. Our results showed the high accuracy of the 3D-printed biliary model in replicating anatomical structures of the biliary system with significant differences in measurement between the STL file and 3D-printed model. The significant discrepancy in measurements could be due to inconsistencies among the orientation and location of anatomical landmarks between post-processed data (STL file) and 3D-printed physical models and this needs to be considered in future studies [82]. The large discrepancy was also reported by Bati et al., with significant differences in dimensional measurements between 3D STL images and the original images/images of the 3D-printed model [80]. A 3D-printed model of the biliary system can be used for education and training, as well as the treatment of complex biliary disease [83,84].

### 5.6. 3D-Printed Chest Models

**Figure 22:**
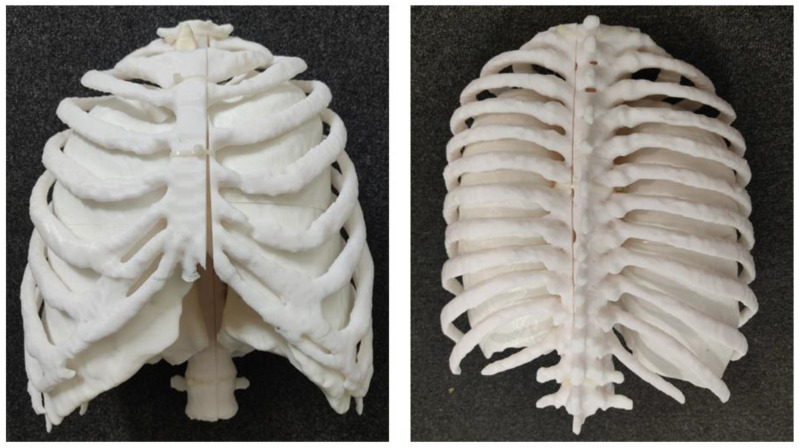
3D-printed chest model comprising three components: Lung shells, thoracic ribs, thoracic vertebrae and trachea (frontal and posterior views).

We printed a chest phantom comprising lungs, trachea, ribs and thoracic vertebrae to provide a realistic anatomical environment to host 3D-printed models such as heart, coronary artery and pulmonary artery models. This is especially important for studying optimal CT scanning protocols, with a reduction in radiation dose, with 3D-printed models placed in a thoracic cavity with anatomical structures surrounding them (Figure 22). Our previous studies showed the feasibility of using 3D-printed aorta, coronary and pulmonary artery models to determine appropriate CT protocols with lower radiation doses but acceptable image quality. However, these phantoms were placed in a simple plastic or acrylic container without having anatomical thoracic or abdominal structures available [53,55,62,85,86]. Our developed 3D-printed chest model could further advance our previous research with robust findings generated, as all of the anatomical structures are simulated in the 3D-printed models.

### 5.7. 3D-Printed Models of Abdominal and Pelvic Organs

**Figure 23:**
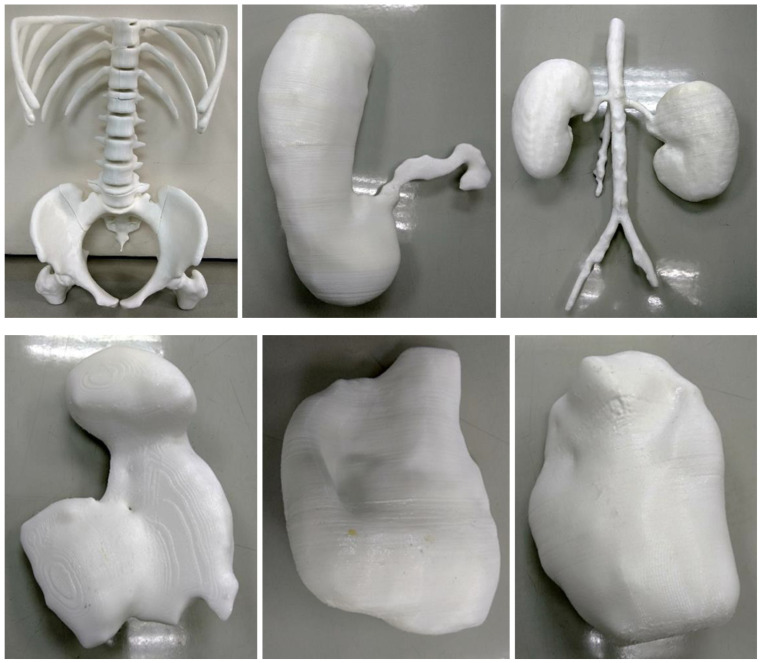
3D-printed models of abdominal organs except for the small and large intestines. Top row from left to right: 3D-printed skeleton, stomach, kidneys and abdominal aorta models. Bottom row from left to right: 3D-printed spleen, bladder and uterus models. Reprinted with permission from Etherton et al. [88].

**Figure 24:**
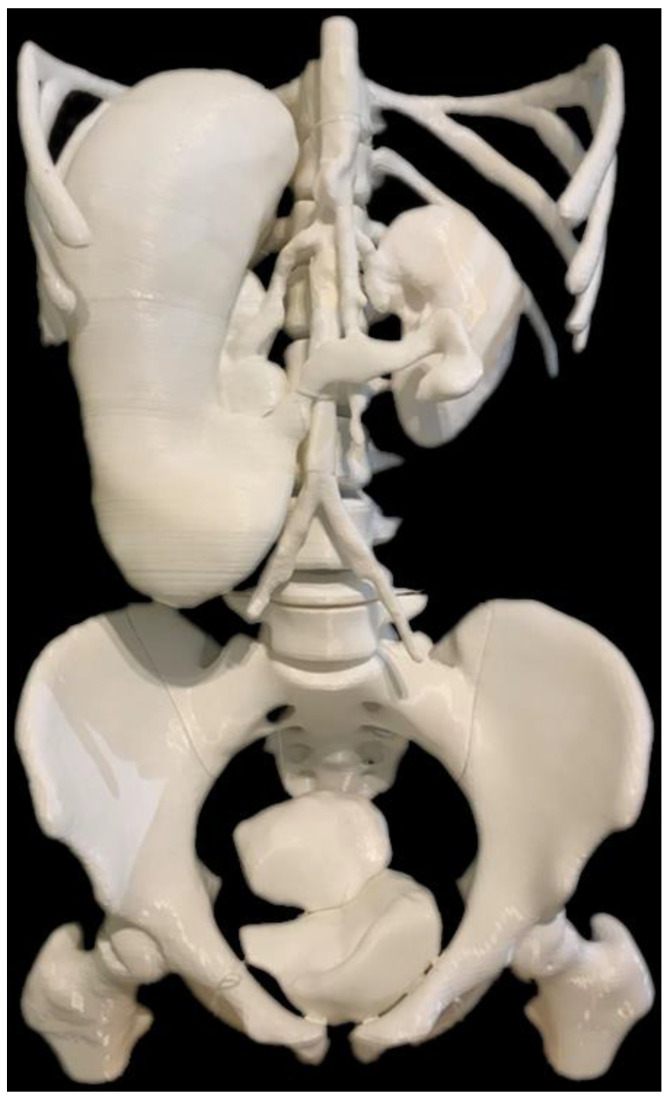
Demonstration of 3D-printed models of abdominal organs after being assembled together. Reprinted with permission from Etherton et al. [88].

We encountered a case of situs ambiguus which is a rare congenital anomaly with multiple abdominal or pelvic organs abnormally positioned [87]. Both CT and MRI images were used to segment abdominal organs including the liver, spleen, stomach, kidneys, aorta and its main arterial branches, bladder, and uterus [88]. These organs were 3D-printed for a demonstration of the abnormal position of some organs within the abdominal and pelvic regions, as shown in Figure 23 and Figure 24. The 3D-printed models can be used for educational purpose for medical students, family members of the patient and also between clinical colleagues. Personalized 3D-printed models of these abdominal and pelvic organs are shown in a range of applications, from medical education to training and the simulation of surgical procedures to residents and surgical teams, with an improvement in surgery outcomes [89,90,91,92].

## 6. Limitations and Future Research Perspectives

In this article, we share our experience of printing a number of low-cost, affordable personalized models with different medical applications, which range from medical education to preoperative planning and the simulation of surgical procedures, as well as the development of optimal CT scanning protocols in cardiovascular imaging. More than half of the models are used in cardiovascular research area with a focus on the investigation of a 3D-printed CHD model, which is most commonly reported in the literature, while other models have been applied to create different tumours for preoperative planning purpose.

TPU is the most common material used in the production of our models with an average cost of less than USD 100, which is much cheaper than models printed with high-cost materials. The average cost of our models is similar to Gomez-Ciriza’s experience of printing affordable heart models [93]. Our results have shown the high accuracy of these 3D models with excellent agreement in dimensional measurements between original images (mainly CT images), 3D-printed models and STL files. 3D-printed CHD models serve as a valuable educational tool when studying cardiac anatomy and pathology and this is well explored by our studies and others [30,31,32,33,34,35,36,39,40]. Despite these promising results, our 3D-printed models are limited in that they use a limited range of materials which could affect their application in other areas, such as simulating interventional cardiology or radiology, or surgical procedures, as these applications require the models to be printed with soft and elastic materials [58,94,95]. Users or operators prefer to have the models printed with tissue-mimicking materials similar to normal human tissues so that they will acquire a similar tactile experience when performing these simulation procedures on the 3D-printed models [96]. Thus, exploring the use of new printing materials including biocompatible materials to print more realistic models is our ongoing research direction. Printing time is quite lengthy for some of the models (>100 h) and this could be reduced with the improvement in 3D-printing technology in the near future.

Another limitation of our 3D-printed models (heart and vascular models) is their static nature, without having functional capability. These 3D-printed heart and vascular models are acceptable for educational purposes; however, when used for the simulation of physiological changes in the cardiovascular system, a functional model is desirable. This could be overcome by connecting 3D-printed models to a cardiac pump as indicated by some studies [59,97]. To generate high-quality 3D-printed heart and vascular models, the quality of the original cardiac images determines the creation of 3D models. This is especially challenging in younger children due to body movement or inappropriate timing between breaths. This could be minimised by using ECG-gated, multi-beat studies to acquire high-resolution images. Furthermore, multiple image registrations such as combining models from 3D echocardiography with those from cardiac MR images may allow the acquisition of complete cardiac models [98].

A comparison of VR, AR and mixed reality (MR) with 3D-printed models for medical applications is another area that we are currently exploring as these innovative 3D visualization tools have shown great potential in both education and preoperative planning. VR/AR/MR could complement 3D-printed models for some applications, given the superior advantages of creating an immersive virtual environment, and the interaction between virtual objects and the real environment [99,100,101,102,103,104,105]. Thus, the use of these latest technologies will further advance medical education and clinical practice. Table 4 summarises the challenges and limitations in printing these models based on our experience. With further improvements in 3D-printing technology, it is expected that these limitations will be overcome in the near future.

## 7. Conclusions

We have demonstrated our experience of utilizing a 3D-printing facility to print a number of personalized models and shared our results of their educational and clinical value through the use of these 3D-printed models. These models are printed with different materials showing the accuracy of the physical models and acceptability of these models for various applications. 3D-printed models are of great value in the education of medical students or graduates, patients or patients’ families to enhance their understanding of anatomy and pathology, as well as disease condition. 3D-printed personalized models have shown clinical value in assisting preoperative planning and the simulation of complex or challenging surgical procedures, with improved clinical outcomes by reducing risks or complications associated with operations. Furthermore, realistic 3D-printed physical modes can be used as a training tool for residents or junior doctors to develop their confidence and clinical skills prior to operating on patients. Our 3D-printing experience has laid a good foundation for the further development of 3D-printing technologies and the use of advanced or new materials to enable the printing of more realistic models with beneficial outcomes to improve our education and patient care.

## Figures and Tables

**Table 1:** List of 3D-printed models that were printed with different printers and materials for medical applications.

Anatomical Region	Number of Models	Original Data Source	Applications of 3D Printed Models	3D Printer/Printing Materials/Costs	3D Printing Parameters (Resolution, Printing Time)
Cardiovascular system					
Heart	4	CT	Congenital heart disease for education and preoperative planning	Printer: Anycubic Photon S Material: Polyurethane (PU) 80A Cost: USD 25 per model	Model was printed at a resolution of 47 μm for the *x*- and *y*-axis planes; 10 μm for *z*-axis planes Time: ~10 h per model
Coronary artery	6	CT	Coronary stenosis for optimal CT protocols	Printer: Anycubic Photon S Material: Polyurethane (PU) 80A Cost: USD 15 per model	Model was printed at a resolution of 47 μm for the *x*- and *y*-axis planes. 10 μm for z-axis planes Time: ~6 h per model
Calcified plaque	22	N/A	For the simulation of calcified plaques	Printer: The mould (circular rod) was printed with polylactic acid (PLA) using Ultimaker 2 + Extended Material: Silicone + 32.8% calcium carbonate Cost: USD 10 for the mould	The mould was printed at a resolution of 12.5 μm for the *x*, *y* and *z*-axis planes. Time: ~3 h for the mould
Aorta	6: Abdominal aortic aneurysm: 5 Aortic dissection: 1	CT	Aortic aneurysm and aortic dissection for the simulation of endovascular repair and CT protocols	Printer: Ultimaker 2+ Extended/Raise3D N2 Plus Materials: Thermoplastic polyurethane (TPU) 95A, PLA, polyethylene terephthalate glycol (PETG), polymethacrylate (PMMA) and high impact polystyrene (HIPS) Cost: USD 50 per model	Aorta was printed with PLA, HIPS, PMMA were at a resolution of 12.5 μm for the *x*, *y* and *z*-axis planes. Aorta was printed with TPU95A was at a resolution of 12.5 μm for the *x* and *y*-axis planes; 10 μm for *z*-axis plane Time: ~100 h per model
Tumours					
Breast	1	MRI	Breast cancer model for breast MRI protocols	Printer: Breast skin shell was printed using Raise3D N2 Plus; Fibroglandular tissues were printed using Anycubic Photon S Materials: Breast skin shell was printed PLA; Fibroglandular tissues were printed using Magma H LINE Photopolymer Resin Cost: USD 30 for breast skin shell and USD 25 for fibroglandular tissues	Breast skin shell was printed at a resolution of 12.5 μm for the *x* and *y*-axis planes; 10 μm for *z*-axis plane Fibroglandular tissues were printed at a resolution of 47 μm for the *x*, *y* and *z*-axis planes Time: ~40 h for breast skin shell and 50 h for fibroglandular tissues
Biliary cyst	1	CT	Accuracy and preoperative planning	Ultimaker 2+ Extended Material: TPU 95A Cost: USD 35	Model was printed at a resolution of 12.5 μm for the *x*, *y* and *z*-axis plane Time: ~70 h
Pancreas	2: Pancreatic tumour: 1 Abdominal aorta and branches: 1	CT	Pancreatic cancer for preoperative planning and education	Printer: Abdominal aorta and arterial branches were printed using Anycubic Photon S. Pancreatic tumour was printed using Raise3D N2 Plus. Materials: Abdominal aorta and arterial branches were printed with PU80A; Pancreatic tumour was printed with PLA Cost: USD 20	Abdominal aorta and arterial branches were printed at a resolution of 47 μm *x*, *y* and *z*-axis planes. Pancreatic tumour was printed at resolution of 12.5 μm *x*, *y* and *z*-axis planes Time: ~20 h
Kidneys	1	CT	Renal cell carcinoma for preoperative planning	Ultimaker 2+ Extended Material: TPU 95A Cost: USD 20	Model was printed at a resolution of 12.5 μm for the *x*, *y* and *z*-axis planes Time: ~70 h
Others (thoracic and abdominal organs)					
Chest (lungs, thoracic vertebral column and ribs)	1 with three compoents: lung shell, thoracic ribs/vertebrae and trachea	CT	Creation of anatomical environment for cardiovascular imaging studies	Printer: Thoracic ribs and lung shell were printed using Raise3D N2 Plus; Trachea was printed using Ultimaker 2+ Extended Materials: Thoracic ribs/vertebrae and lung shell were printed with PLA Trachea was printed with TPU 95A. Cost: USD 75 for thoracic ribs and lung shell; USD 10 for trachea	Thoracic ribs and lung shell were printed at a resolution of 12.5 μm for the *x* and *y*-axis planes; 10 μm for *z*-axis plane Trachea was printed at a resolution of 12.5 μm for the *x*, *y* and *z*-axis planes Time: ~450 h for thoracic ribs and lung shell; ~17 h for trachea
Abdomen and pelvis	Stomach: 1 Kidneys: 1 Spleen: 1 Bladder: 1 Uterus: 1 Skeleton: 1	CT	Multiple organs for a case of situs ambiguus	Printer: Skeleton was printed using Raise3D N2 Plus (Raise3D, USA) Other organs were printed using Ultimaker 2+ Extended (Ultimaker BV, Netherland) Material: Skeleton: PLA Other organs: TPU 95A Cost: USD 55 for skeleton and USD 75 for other organs	Skeleton was printed with a resolution of 12.5 μm for the *x* and *y*-axis planes and 10 μm for *z*-axis plane Other organs were printed at a resolution of 12.5 μm for the *x*, *y* and *z*-axis plane Time: Skeleton: ~250 h Other organs: ~250 h

**Table 2:** 3D-printed heart model accuracy in comparison with original source images according to the current literature. Modified from Lee et al. [22].

Studies Reporting Accuracy Comparison	No. of Models Printed	Comparisons	Mean Difference (mm)	Analysis Method
Lee et al. [22]	3	3D model vs. original CT 3D model vs. CT of 3D model 3D model vs. STL files Original CT images vs. STL files	0.21 ± 0.37 mm −0.11 ± 0.47 mm 0.1 ± 0.28/ 0.17 ± 0.48 mm 0.12 ± 0.23/ 0.12 ± 0.25 mm	Pearson’s correlation/ Bland–Altman plot
Valverde et al. [23]	40 (20 selected for accuracy comparison)	3D model vs. both CT and MRI 3D model vs. original CT 3D model vs. original MRI	0.27 ± 0.73 mm −0.16 ± 0.85 mm −0.30 ± 0.67 mm	Bland–Altman plot
Olejník et al. [24]	8	CT images vs. STL	0.19 ± 0.38 mm	Bland–Altman plot
		3D model vs. in vivo	0.13 ± 0.26 mm	
Olivieri et al. [25]	9	3D model vs. echocardiography	0.4 ± 0.9 mm	Pearson’s correlation/ Bland–Altman plot
Lau et al. [26]	1	3D model vs. CT	0.23 mm	Pearson’s correlation
Mowers et al. [27]	5	2D echo vs. digital 3D	0 mm	Pearson’s correlation/ Bland–Altman plot
		2D echo vs. 3D model	0.3 mm	
Parimi et al. [36]	5	3D model vs. rotational angiography	No significant difference between 3D models and biplane angiography measurements (*p* = 0.14)	Pearson’s correlation/ Bland–Altman plot

DICOM—digital imaging and communications in medicine, CT—computed tomography, MRI—magnetic resonance imaging, STL—standard tessellation language.

**Table 3:** Subgroup analysis for participants’ responses on the ratings for VR and 3DPHM.^a^ Reprinted with permission under the open access from Lau et al. [52].

Question		Doctors’ Group, *n* = 9	Non-Doctors’ Group, *n* = 20	Mann–Whitney U-Value	*p*-Value
	Option				
Rate the usefulness of VR models in medical education		4 (11.22)	4 (16.70)	56.00	0.07
Rate the usefulness of 3DPHM in medical education		4 (11.11)	5 (16.75)	55.00	0.07
Rate the usefulness of VR models in pre-operative planning		4 (11.06)	4 (16.77)	54.50	0.07
Rate the usefulness of 3DPHM in pre-operative planning		4 (12.39)	4.5 (16.18)	66.50	0.23

3DPHM—3D-printed heart models; VR, virtual reality. ^a^ Data are median score (mean rank).

**Table 4:** Limitations and challenges of 3D printing for medical applications.

3D Printing Applications	Challenges and Limitations	References
Pre-surgical planning and simulation (cardiac disease and tumours)	Limited choices of printing material to simulate the required tissue properties (both radiological and mechanical properties). Limitations in multi-colour and multi-material 3D printing to delineate tumour from the normal tissue. Time-consuming post-processing of the printed model (to remove the support structures of the model).	[26,31,33,37,50,52,72,78,82]
Simulation of interventional cardiac/radiological procedures	Limited choices of printing material to simulate the required tissue properties (radiological properties). The smaller printing size of the 3D printer has limited the ability to print the model in a whole piece. The model will need to be printed in smaller pieces and joined together, thus increasing the post-processing time.	[33,34,53,54,55,61,64,66,67,85,86]
Medical education	Limitations in multi-colour and multi-material 3D printing to delineate different tissues in the model. Limited choices of printing material to produce a realistic 3D model with mechanical properties similar to human tissues.	[49,52,88]
Patient/family education/communication	The FDM and DLP 3D printers are limited by lower printing speed and smaller printing size. This has limited the application of 3D-printed models for patient/family education as the printing involves higher human resources and longer duration.	[88,104]

FDM—fused deposition modelling, DLP—digital light processing.

## Data Availability

Not applicable.
